# Perinephritic Abscess; Operation, Etc.

**Published:** 1888-04

**Authors:** Rudolph Menger

**Affiliations:** San Antonio, Texas


### PERINEPHRITIC ABSCESS.—OPERATION, ETC.

*By Dr. Rudolph Menger, San Antonio, Texas.*

For Daniel's Texas Medical Journal.

DR. Wm. BOLL, of Castroville, Texas, is treating a very interesting case of perinephritic abscess, the history of which is, as reported to me by Dr. Boll, as follows :

" A farmer, aged 39, complained of pain in the right lumbar region, after several small furuncles had made their appearance along the back and apparently healed up. Pulse, temperature, etc., normal. On examination a slight protuberance can be detected through percussion in the right lumber region, reaching toward and near the margin of the ileum and toward the umbilical region. On palpation a deep seated pain is noticed. Percussion of the liver normal, also urine perfectly normal. After two weeks application of counter irritants, iodine and warm poultices, and internally iodide of potassium, the tumor gradually diminished in size and form a great deal. But the patient complained now of more pain in the back running along the psoas muscle toward the right leg. Signs of high fever, rapid emaciation and general uneasiness were now manifested. The temperature runs up to 105 ° ; pulse weak and very frequent. I now searched for an abscess cavity with the aspirator needle, inserting the same from the front, as at no time could any fluctuation be established. After this I also punctured from the back, one inch from the twelvth rib, three inches from the spinal column. but it was impossible to aspirate anything into the aspirating bottle, because, as proven later, the pus was so tough that it would not pass the needle, although some pus had advanced so as to be clearly recognized through the glass tube adjustment.

Thus far goes Dr. Boll's statement of the case. I was summoned by him in consultation and to assist him in opening the cavity thoroughly under chloroform. After having the patient thoroughly prepared for the occasion and using a spray atomizer and the greatest possible cleanliness, we first aspirated again below the last rib, close to the spinal column, and succeeded at once to tap a small quantity of thick puss off. Then we cut at this same point down to the region of the kidney, and succeeded, after some difficulty, to strike the pus cavity about three inches from the surface, at least we had to insert the entire index finger to reach the cavity which seemed to be filled with thick doughy pus, tissue detritus and adhesions, and I could plainly feel the lower margin of the kidney surrounded with softened tissue and pus. The cavity was then widened and thoroughly cleaned out with corrosive sublimate solution, and a medium sized drainage tube inserted as far back as possible. The wound around the tube was stitched and dressed with iodoform cotton and antiseptic bandages. I may state that there was high septic fever during the night before the operation, and that a decrease of temperature set in a short while after the operation, falling somewhat below normal, to 97,25, and after that remained quite normal, varying between 99° and 101°. According to Dr. Boll's last report, now the 12th day after the operation, the wound is healing per primam and the drainage tube working well and the patient is in far better condition.

The above case is noteworthy regarding the state of the urine, which at all times was normal, the constant severe pain and increased respiration indicating undoubtedly septic infection. In taking a deep inspiration, whilst coughing and even during a difficult evacuation of the bowels, the pain was also increased. And the case was also a very interesting one in regard to the *differential diagnosis*. There were no constant symptoms of hepatitis, the liver being perfectly normal, although with the onset of the trouble the patient did have a slight icterus, and by palpation and percussion no tumor indicating perihepatitic abscess could be established with any certainty, and as to typhlitis or perityphlitis or pyothorax, the same could also be well excluded, taking the entire history of the case into consideration.

